# Knowledge, attitude and practice of pharmacists on medication therapy management: a survey in Hospital Pulau Pinang, Penang, Malaysia

**DOI:** 10.1186/s40780-019-0131-9

**Published:** 2019-01-10

**Authors:** Noor Kifah Al-Tameemi, Azmi Sarriff

**Affiliations:** Discipline of Clinical Pharmacy, School of Pharmaceutical Sciences, Universiti Sains Malaysia, Pinang, Malaysia

**Keywords:** Clinical pharmacy, Pharmacy practice, Medication therapy management, Medication therapy adherence clinic, Pharmaceutical services, Hospital pharmacy practice

## Abstract

**Background:**

Medication therapy management (MTM) service provides set of clinical activities to optimize therapeutic outcomes for patients. It requires the collaboration between patient, pharmacist and other healthcare providers to ensure safe and effective use of medicines. The objective of the current study was to assess Hospital Pulau Pinang pharmacists’ knowledge, attitude and practice on MTM service.

**Methods:**

A self-administrated validated survey was carried out among all pharmacists working at Hospital Pulau Pinang.

**Results:**

A total of 93 pharmacists out of 130 (71.5%) were included in the study (61.3% between the age of 20–30 years old, 74.2% female, 68.8% Chinese, 88.2% holding bachelor’s degree and 48.4% working in medication therapy adherence clinic and outpatient pharmacy). Majority of pharmacists had a high level of knowledge and positive attitudes regarding MTM service. All pharmacists agreed that MTM service could improve the quality of health services and most pharmacists were interested in providing MTM service (92.5%). Moreover, 95.7% were interested in acquiring more information about MTM service. About the barriers that might affect MTM service implementation, the most common barriers identified by pharmacists were lack of training (88.2%), need of high budget to implement MTM service (51.6%) and lack of time (46.2%).

**Conclusions:**

Overall, the research findings provide some insights about the Hospital Pulau Pinang pharmacists’ knowledge, attitude and practice regarding MTM service. Majority of pharmacists agreed and showed their interest towards the implementation of MTM service.

## Background

Medication therapy management (MTM) service is defined as a service or group of services that optimize therapeutic outcomes for individual patients [1, 2]. It requires the collaboration between patient, pharmacist, physician and other healthcare providers to control patient’s condition, prevent drug related problems and ensure safe and effective use of medicines to reach patient’s therapy outcomes [3, 4].

In 2003, MTM service was included in Medicare Modernization Act (MMA), United States America (USA), and that was the initial step to start applying MTM service at different healthcare sectors. MMA identified the importance of implementing MTM service to achieve three different goals: enhance patients’ adherence to their medications, give optimal education and counselling to patients regarding their drug therapies and detect any adverse drug reaction associated with the therapy. These goals could be achieved when pharmacists work with physicians and other healthcare professionals to optimize medications use according to national and international guidelines [5].

American Pharmacists Association (APhA) and National Association of Chain Drug Stores Foundation (NACDS) descried five main elements of MTM service including: Medication Therapy Review (MTR), Personal Medication Record (PMR), Medication Related Action Plan (MAP), Intervention or Referral and Documentation and Follow-Up. These five elements are needed to implement MTM service in pharmacy practice [6].

MTM service can be also sorted into levels to make performing of this service more scalable and applicable. There are three levels of MTM service. First level is Adherence Management, and this is the lowest level. In this level pharmacists ensure that patients are adhering to the medications that they take for certain type of disease such as; hypertension, diabetes or dyslipidemia. Pharmacists need general clinical knowledge to apply this level. The importance of Adherence Management is to enhance health outcomes and reduce costs linked to lack of adherence. The second level is Interventions on Drug-Related Problems. In this level pharmacists apply Medication Therapy Review Service (MTRS) to ensure safe and effective use of all medications that are used by patients. There are two types of MTRS: Comprehensive and Targeted. In Comprehensive type, pharmacists review all medications taken by patients including prescribed, non-prescribed, over the counter and herbal medications. Then pharmacists detect if there is any problem associated with these medications and try to resolve it. In Targeted MTRS, pharmacists assess specific actual drug related problem. This happens after providing comprehensive service first, so pharmacists already have information about all medications taken by patients. The third and highest level of MTM service is Disease State Management Service. In this level pharmacists cover all chronic diseases that patient is diagnosed with. Pharmacists participate not only in drug related problem interventions, but also in non-drug therapy, lifestyle modifications and other activities that optimize health outcomes [7].

In Malaysia, Ministry of Health (MOH) established medication therapy adherence clinic (MTAC) in 2004 to be the first pharmacist-managed clinic in Malaysia. MTAC was implemented to enhance patients’ adherence to their medications. Over the past years, many types of MTACs has been introduced and covered many diseases and conditions such as; diabetes, stroke and respiratory disease. In 2013, thirteen types of MTACs were established by MOH with 660 different facilities offering the service around Malaysia [8–10].

The main difference between MTM service and MTAC is that MTAC focuses mainly about adherence. The main objective of this service is to improve patients’ understanding of medicines taken by them. On the other hand, MTM service includes furthermore services such as; detection of any adverse drug reaction associated with the therapy taken by the patient and disease state management [7, 10].

## Methods

### Aims of the study

(i) Assess the level of knowledge, attitude and practice (KAP) of medication therapy management service among pharmacists working in Hospital Pulau Pinang.

(ii) Identify various challenges and barriers towards providing MTM service in the future.

### Study setting and design

This study was a cross-sectional survey of medication therapy management service KAP and was carried out at Hospital Pulau Pinang. Questionnaires were distributed among all pharmacists that were working at the hospital during research period, between March 2018 and July 2018.

### Study participants

A convenience sample of 130 pharmacists was included in the study. All trainee pharmacists and those who were on leave during research period were excluded.

### Development of questionnaire

After referring to many studies regarding MTM service in the literature, a structured questionnaire was designed to cover all the main key points of the research and in a way that suit with local population of Penang. Later, the questionnaire was validated by six lecturers from the discipline of clinical pharmacy at Universiti Sains Malaysia and it was examined for content relevance and appropriateness. Minor modifications were done based on their comments. Moreover, face validity was done by giving the questionnaire to a group of five pharmacists working at Hospital Pulau Pinang. Then the final structure of the questionnaire was conducted based on all comments given by participants. Finally, for reliability, a pilot study was done on 15 pharmacists working at Hospital Pulau Pinang. The pharmacists who were participated in pilot study have been excluded from final analysis. The survey was written in English and contains 34 items of questionnaire distributed between four sections. Table 1 describes each section in detail.

**Table 1:** Description of each section in the survey

Section title	Description of the section
Section 1: sociodemographic characteristics	This section consisted of six questions including data about gender, age, race, pharmacy practice setting, educational degree and years of experience.
Section 2: knowledge of pharmacists about MTM service ^a^	Ten questions were included in this section. These questions were either multiple choice questions or true/false questions. Each correct answer was given “1” point while wrong answer was given “0” point. Then the sum score was calculated, from minimum sum of “0” to maximum sum of “10”, for each responder. Finally, the scores were classified into three levels as follows: 1. high level: 10–8 scores; 2. moderate level: 7–6 scores; and 3. low level: 5–0 scores.
Section 3: attitude of pharmacists on MTM service ^b^	This section contained 6 positive statements and 5-point Likert scale was used to assess the attitude. Each question contained five statements and the rating scale was measured as follows: positive statement with choices strongly agree, agree, neutral, disagree, and strongly disagree and scores 5, 4, 3, 2, and 1, respectively.
Section 4: practice of pharmacists on MTM service ^c^	This section consisted of 12 questions used to assess the practice of pharmacists on MTM service, the compliance and state the barriers that might affect providing MTM service in hospital setting. All questions were Yes/No questions. No answer was given “0” point and Yes answer was given “1” point.

*MTM* medication therapy management

^a^Cronbach’s alpha for knowledge part was 0.326. To classify the scores of knowledge into levels, the original Bloom’s cut-off points, 100–80.0%, 79.0–60.0%, and ≤ 59.0%, were adapted from KAP study done to measure knowledge, attitude, and practice of the food safety towards compliance with abattoir laws between the workers in Terengganu, Malaysia [22]

^b^Cronbach’s alpha for attitude part was 0.716

^c^Cronbach’s alpha for practice part was 0.822

### Data analysis

Statistical Package for the Social Sciences (SPSS), version 23.0, was used for data analysis. Descriptive data was presented in percentage (%) for categorical data and in mean and Standard Deviation (SD) for numerical data. Chi-Square test and Fisher’s Exact test were performed to compare the differences in level of knowledge about MTM service based on sociodemographic characteristics. In addition, Mann Whitney U test and Kruskal Wallis test were conducted to investigate the differences between sum score of KAP and sociodemographic characteristics.

## Results

### Demographic information

A total of 130 questionnaires were distributed and 93 questionnaires were returned with response rate of 71.5%.

Table 3.1 displays the frequency of participants according to sociodemographic characteristics. Most of responders were female (*N* = 69, 74.2%). There were more responders in the age group 20–30 years old (*N* = 57, 61.3%) and majority of pharmacists were from Chinese ethnicity (*N* = 64, 68.8%). The highest number of pharmacists were holding bachelor’s degree (*N* = 82, 88.2%) and most of responders were outpatient pharmacists (*N* = 31, 33.3). Moreover, majority of pharmacists had 0–10 years of experience (*N* = 84, 90.3%) compared with pharmacists that had 11–20 years of experience (*N* = 9, 9.7%) (Table 2).

**Table 2:** Sociodemographic data

Characteristics	N	%
Total	93	100.0
Gender		
Male	24	25.8
Female	69	74.2
Age		
20–30	57	61.3
31–40	36	38.7
Race		
Malay	21	22.6
Chinese	64	68.8
Indian	8	8.6
Degree		
Bachelor’s	82	88.2
Master’s	10	10.8
PhD	1	1.1
Pharmacy Practice Setting		
Inpatient pharmacist	20	21.5
Outpatient pharmacist	31	33.3
MTAC pharmacist	14	15.1
Clinical pharmacist	12	12.9
Pharmacist working at drug information center	7	7.5
Pharmacist working at store	6	6.5
Pharmacist working at total parenteral nutrition preparation	3	3.2
Years of experience		
0–10	84	90.3
11–20	9	9.7

*MTAC* medication therapy adherence clinic

### Knowledge of pharmacists about medication therapy management service

Ten different questions were used to assess pharmacists’ knowledge regarding MTM service (Table 3). The first five questions were about MTM service and the other questions were about MTAC. Each correct answer was given 1 mark with a total of 10 marks. The median of total score of knowledge was equal to 9. The minimum score obtained was equal to 6 and maximum score reported was equal to 10. Generally, majority of responders could answer most of the questions correctly which indicated that pharmacists had very good knowledge about MTM service.

**Table 3:** Pharmacists’ knowledge regarding medication therapy management service

Questions	N (%)	
	Correct answer	Incorrect answer
1- MTM is defined as service or group of services that: optimize therapeutic outcomes for individual patients	67 (72.0)	26 (28.0)
2- Core elements of MTM service are Medication Therapy Review (MTR), Personal Medication Record (PMR), Medication Related Action Plan (MAP), Intervention or Referral and Documentation and Follow-Up	85 (91.4)	8 (8.6)
3- Medication therapy management services have three goals which are: to improve the understanding medication uses, medication adherence and detection of medication related problems	89 (95.70)	4 (4.3)
4- Any patient who uses prescription and nonprescription medications, herbal products or other dietary supplements could potentially benefit from MTM service	82 (88.2)	11 (11.8)
5- Primary role of MTM service is aid with adherence and disease state management	93 (100.0)	0 (0.0)
6–13 types of MTAC have been implement by the Ministry of Health	49 (52.7)	44 (47.3)
7- MTAC helps in maximizing the benefits of medications and assessing therapy outcome	90 (96.8)	3 (3.2)
8- MTAC can diminish emergency room visits of patients and decrease total healthcare costs of chronic diseases	89 (95.7)	4 (4.3)
9- MTAC can promote patient’s satisfaction	92 (98.9)	1 (1.1)
10- MTAC is a good information resource for patients	93 (100.0)	0 (0.0)

*MTM* medication therapy management; *MTAC* medication therapy adherence clinic

Table 4 shows the level of knowledge of participants regarding MTM service. Majority of participants were considered with high level of knowledge (*N* = 86, 92.5%) compared to those with moderate level of knowledge (*N* = 7, 7.5%). While none of the pharmacists considered with low level of knowledge.

**Table 4:** Distribution of knowledge level regarding medication therapy management service

Level	N	%
Total	93	100.0
High (8–10 Scores)	86	92.5
Moderate (6–7 Scores)	7	7.5
Low (0–5 Scores)	0	0.0

Minimum score = 0; Maximum score = 10; Median = 9

Apart from high level of knowledge (Table 5), higher knowledge regarding MTM service existed in female, younger pharmacists in age group 20–30 years old, Indian, pharmacists holding PhD degree and had 11–20 years of experience. All pharmacists working at MTAC, drug information center, total parenteral nutrition preparation and clinical pharmacists were with high level of knowledge and half of responders working at store were with high level of knowledge. There was a significant association between the level of knowledge and pharmacy practice setting (*P* = 0.004).

**Table 5:** The level of knowledge about medication therapy management service based on sociodemographic characteristics

Characteristics	Knowledge level (%)		*P*-Value
	Moderate	High	
Total	7 (7.50)	86 (92.5)	
Gender			0.469
Male	1 (4.2)	23 (95.8)	
Female	6 (8.7)	63 (91.3)	
Age			0.815
20–30	4 (7.0)	53 (93.0)	
31–40	3 (8.3)	33 (91.7)	
Race			0.336
Malay	3 (14.3)	18 (85.7)	
Chinese	4 (6.2)	60 (93.8)	
Indian	0 (0.0)	8 (100.0)	
Degree			0.916
Bachelor’s	6 (7.3)	76 (92.7)	
Master’s	1 (10.0)	9 (90.0)	
PhD	0 (0.0)	1 (100.0)	
Pharmacy Practice Setting			0.004*
Inpatient pharmacist	1 (5.0)	19 (95.0)	
Outpatient pharmacist	3 (9.7)	28 (90.3)	
MTAC pharmacist	0 (0.0)	14 (100.0)	
Clinical pharmacist	0 (0.0)	12 (100.0)	
Pharmacist working at drug information center	0 (0.0)	7 (100.0)	
Pharmacist working at store	3 (50.0)	3 (50.0)	
Pharmacist working at total parenteral nutrition preparation	0 (0.0)	3 (100.0)	
Years of experience			0.368
0–10	7 (8.3)	77 (91.7)	
11–20	0 (0.0)	9 (100.0)	

*MTAC* medication therapy adherence clinic; ^*^P < 0.05, Significant

### Attitude of pharmacists towards medication therapy management service

Table 6 shows that majority of the pharmacists agreed with most of the statements on attitude towards MTM service. High proportion of responders (92.5%) believed that pharmacist’s role is more than dispensing medications and 90.3% of them agreed that patients would receive more information about their chronic diseases and medicines by applying MTM service. 84.9% of pharmacists fully supported MTM service core elements and 79.6% of responders agreed that patient’s health outcomes would be improved when medications are monitored by a pharmacist as compared to other healthcare providers. Majority of pharmacists (91.4%) believed that MTM service requires more knowledge than basic information of pharmacy practice and high percentage of pharmacists (93.5%) agreed that applying MTM service would allow pharmacists to participate in patient care at a broader spectrum.

**Table 6:** Pharmacists’ attitude towards medication therapy management service

Statements	N (%)			Mean (*SD*)
	SA + A	Neutral	D + SD	
1- Besides the processes of normal dispensing functions, reviewing patient’s medication profile and providing interventions are important as roles of pharmacist to prevent adverse effects	86 (92.5)	7 (7.5)	0 (0.0)	4.46 (*0.635*)
2- By applying MTM service, patients would receive adequate and beneficial information about their chronic disease (s) and medication therapies from their providers	84 (90.3)	9 (9.7)	0 (0.0)	4.34 (*0.651*)
3- By considering the five core elements of MTM service: Medication Therapy Review (MTR), Personal Medication Record (PMR), Medication Related Action Plan (MAP), Intervention or Referral, and Documentation and Follow-Up, do you agree that MTM service is valuable?	79 (84.9)	14 (15.1)	0 (0.0)	4.32 (*0.725*)
4- Patient’s health outcomes would be improved when medications are monitored by a pharmacist as compared to other health care providers	74 (79.6)	14 (15.1)	5 (5.4)	4.14 (*0.867*)
5- Applying MTM service requires more knowledge than basic information of pharmacy practice	85 (91.4)	7 (7.5)	1 (1.1)	4.40 (*0.678*)
6- Providing MTM service is a unique opportunity for pharmacists to participate in patient care at a broader spectrum	87 (93.5)	6 (6.5)	0 (0.0)	4.48 (*0.619*)

*MTM* medication therapy management; *SA* Strongly Agree; *A* Agree; *D* Disagree; *SD* Strongly Disagree; *SD Standard Deviation*

### Practice of pharmacists regarding medication therapy management service

Table 7 describes the practice of pharmacists towards MTM service and the barriers that might affect MTM service implementation in the future. All pharmacists agreed that MTM service improves the quality of health services and most pharmacists were interested in providing MTM service in the future (92.5%). Moreover, 95.7% of them were interested in acquiring more information about MTM service. 87 pharmacists had a desire for taking training workshops regarding MTM service and 82 of them believed that online education system is a good way to provide training for pharmacists regarding MTM service.

**Table 7:** Pharmacists’ practice towards medication therapy management service and barriers that affect MTM service implementation

Questions	N (%)	
	Yes	No
1- If MTM service will be implemented in the future, would you like to be an MTM service provider?	86 (92.5)	7 (7.5)
2- Do think implementing MTM service in the future is important to improve the quality of health?	93 (100.0)	0 (0.0)
3- Are you interested in learning more information about providing MTM service?	89 (95.7)	4 (4.3)
4- Is online education a good way to provide training for pharmacists?	82 (88.2)	11 (11.8)
5- Do you prefer live workshops to provide training for pharmacists?	87 (93.5)	6 (6.5)
6- In your current practice, do you think that you spend enough time counseling your patients?	19 (20.4)	74 (79.6)
7- Do you think that you will have enough time to apply MTM service in the future?	50 (53.8)	43 (46.2)
8- Does your pharmacy or the place that you work in currently have a private counseling area?	93 (100.0)	0 (0.0)
9- Do you usually access (online or hard copies) most updated treatment guidelines available for diseases such as hypertension (JNC-VIII) or hyperlipidemia (ATP III Guidelines)?	62 (66.7)	31 (33.3)
10- Do you have an easy access (online or hard copies) to the guidelines and drug information resources such as Micromedex, Clinical Pharmacology or others?	70 (75.3)	23 (24.7)
11- Lack of training is one of the potential barriers regarding applying MTM service in the future?	82 (88.2)	11 (11.8)
12- Do you think that applying MTM services need high budget?	48 (51.6)	45 (48.4)

*MTM* medication therapy management

About the barriers that might affect MTM service implementation, the most common barriers identified by pharmacists were lack of training (88.2%), need of high budget (51.6%), lack of time (46.2%) and difficulty to access current guidelines (24.7%). All pharmacists stated that the physical environment is not a barrier because the hospital contains counselling rooms.

### Comparison of total score of KAP between the grouping variable

Examination of differences in total score of KAP between sociodemographic variables (Table 8) showed that there was a significant difference in total score of KAP between responders that were working at MTAC and outpatient pharmacy compared with pharmacists who were working at other pharmacy practice settings. Pharmacists who were working at MTAC and outpatient pharmacy had higher total score of KAP compared with the other group. Similarly, a significant difference in total score of KAP reported between pharmacists who had 0–10 years of experience compared to those that had 11–20 years of experience and showed that people with more experience had higher total score of KAP.

**Table 8:** The differences in total score of KAP based on sociodemographic variables

Characteristics	Z	*P*-value
Gender	−0.653	0.514
Male (Median: 40.0, IQR: 7)		
Female (Median: 40.0, IQR: 6)		
Age	−0.746	0.456
20–30 (Median: 40.0, IQR: 7)		
31–40 (Median: 41.5, IQR: 6)		
Race	–	0.124
Malay (Median: 42.0, IQR: 6)		
Chinese (Median: 37.5, IQR: 6)		
Indian (Median: 40.0, IQR: 7)		
Degree	−0.066	0.948
Bachelor’s (Median: 40.0, IQR: 6)		
High Degree (Master’s + PhD) (Median: 39.0, IQR: 5)		
Pharmacy practice setting	−2.246	0.025^*^
MTAC + Outpatient Pharmacist (Median: 41.0, IQR: 5)		
Others (Median: 38.0, IQR: 7)		
Years of experience	−2.790	0.005^*^
0–10 (Median: 39.0, IQR: 7)		
11–20 (Median: 43.0, IQR: 2)		

*MTAC* medication therapy adherence clinic; *P < 0.05, Significant

## Discussion

Nowadays, besides dispensing activity, pharmacist’s role has been expanded to include more services such as; patient counseling, enhance patients’ adherence and detect any adverse drug reaction associated with the therapy. Medication therapy management (MTM) service has been implemented to extend pharmacists’ services beyond the regular activities [11]. MTM service is a direct patient care service and although pharmacists are the only healthcare professionals specifically identified as MTM providers in MMA but providing MTM service requires collaboration between all healthcare providers to achieve best therapeutic outcomes for individual patient [12]. A recent review study, that included 21 references, has been conducted to detect the outcomes of pharmacist-nurse collaboration to enhance patients’ adherence to drugs. The results of this study showed that pharmacists and nurses could work together to achieve better outcomes [13].

MTM service becomes one of the potential and valuable clinical pharmacy services; therefore, the present KAP research studied the knowledge, attitude and practice of pharmacists regarding MTM service as well as established the potential barriers that might affect MTM service implementation.

Majority of pharmacists participated in our study were female (*N* = 69, 74.2%), Chinese (*N* = 64, 68.8%), holding bachelor’s degree (*N* = 82, 88.2%), young pharmacists in the age group 20–30 years old (*N* = 57, 61.3%) and had 0–10 years of experience (*N* = 84, 90.3%).

Compared with other international studies, our study showed that high percentage of Hospital Pulau Pinang pharmacists were with high level of knowledge regarding MTM service (*N* = 86, 92.5%). This result gave a good hint regarding the level of pharmacists’ awareness about MTM service. Similarly, In USA, cross-sectional studies showed that most of pharmacists had good knowledge regarding MTM service [14, 15].

Regarding the attitude of pharmacists towards MTM service, as showed in Table 6, most pharmacists were with positive attitudes about MTM service. This finding was in agreement with pervious study, done between all community pharmacists working at Iowa, USA, which showed that 90.1% of pharmacists agreed that MTM service is a valuable service and applying MTM service is an important step to enhance pharmacy practice career. Moreover, 86.2% of responders agreed that applying MTM service would give them good opportunity to give higher level of care to their patients [16]. On average, Hospital Pulau Pinang pharmacists who participated in this study had positive attitudes regarding MTM service, similar to other international studies which concluded that pharmacists generally had positive attitudes regarding MTM service [15–18].

In terms of practice, most pharmacists reported that they have willingness to become MTM service providers in the future. This indicates that pharmacists have interest to improve their current services. All pharmacists participated in our study agreed that MTM service implementation will improve the quality of health services. In a systemic review study regarding “pharmacist-led medication review”, MTM service was defined as one of the services that used to optimize the therapeutic outcomes of patients and enhance the quality of services that were obtained by pharmacists [19].

Our study also identified the potential barriers that might affect MTM service implementation in the future. Four potential barriers to pharmacist provision of MTM service were assessed (1) training, (2) cost, (3) time, and (4) accessibility to current guidelines. This result was different than other studies done in USA, where pharmacists identified time as a major barrier [14, 16, 18, 20, 21]. 79.6% of Hospital Pulau Pinang pharmacists stated that they do not spend enough time with their patients and 53.8% of them think that they will have time to apply MTM service in the future. This finding indicates that around half of the pharmacists believed that MTM service will allow them to spend more time with their patients since majority of them do not give sufficient time to their patients because their role in the current practice is limited and include only the regular activities. Applying MTM service in the future will allow pharmacists to connect more with their patients because pharmacists will need to control all patients’ conditions and to collaborate with other healthcare workers to take the right decisions. Spending adequate time with patients will help pharmacists to be more involved in patients’ care plan and to give the optimal instructions to their patients and this will increase patients’ adherence to their medications and minimize hospitalization. Moreover, pharmacists were not reporting physical environment as a potential barrier because Hospital Pulau Pinang contains counseling rooms.

Regarding the differences in total score of KAP between sociodemographic variables, Table 8 showed that there was a significant difference in total score of KAP between responders that were working at MTAC and outpatient pharmacy compared with pharmacists who were working at other pharmacy practice settings as well as significant difference in total score of KAP reported between pharmacists who had 0–10 years of experience compared to those that had 11–20 years of experience. People who were working at MTAC and outpatient pharmacy were having higher total score of KAP comparing to pharmacists working at other places. This indicates that those pharmacists were more familiar with MTM service than others and that could be because they have direct contact with patients and they provide some of direct-patient services such as patients counseling service, giving patients additional pharmacological and nonpharmacological information regarding their diseases and therapies and enhance patients’ adherence to drugs. In addition, our study finds that pharmacists with more experience had higher score of KAP than others with less experience.

### Strength and limitations

This is the first cross-sectional study conducted in Malaysia regarding medication therapy management. On the other hand, there were several limitations that appeared with the current study. Cronbach’s alpha value for knowledge part was equal to 0.326 and this value is low compared to Cronbach’s alpha values for attitude and practice parts. Furthermore, this study was conducted only at one hospital in Penang; so, the results of this study cannot extrapolate to predict the knowledge, attitude and practice regarding MTM service among all Malaysian pharmacists.

### Recommendations

This study found that pharmacists had high level of knowledge and positive attitudes regarding MTM service, so further studies at other hospitals around Malaysia is recommended to take bigger picture about implementing MTM service in Malaysia. In addition, majority of pharmacists were interested to apply MTM service as well as they would like to know more information about MTM service, so we recommend doing many online and live workshops regarding MTM service and to enhance the knowledge of pharmacists regarding this service.

## Conclusions

In conclusion, the research findings provide some insights about the Hospital Pulau Pinang pharmacists’ knowledge, attitude and practice regarding MTM service. Most of pharmacists were with high level of knowledge regarding MTM service and showed positive attitudes about MTM service. In addition, Majority of them had willingness to become MTM service provider in the future and were interested to know more information about MTM service. Moreover, participants recorded lack of training and requirement of high budget as the most common barriers that might affect implementation and provision of MTM service in the future.

## Abbreviations

APhA: American Pharmacists Association
KAP: Knowledge, Attitude and Practice
MAP: Medication Related Action Plan
MMA: Medicare Modernization Act
MOH: Ministry of Health
MREC: Medical Research and Ethics Committee
MTAC: Medication Therapy Adherence Clinic
MTM: Medication Therapy Management
MTR: Medication Therapy Review
NACDS: National Association of Chain Drug Stores
PMR: Personal Medication Record
SD: Standard Deviation
SPSS: Statistical Package for the Social Sciences
USA: United States America

## References

[1] McGivney MS, Meyer SM, Duncan–Hewitt W, Hall DL, Goode JV, Smith RB (2007). Medication therapy management: its relationship to patient counseling, disease management, and pharmaceutical care. Journal of the American Pharmacists Association.

[2] Bunting BA, Cranor CW (2006). The Asheville project: long-term clinical, humanistic, and economic outcomes of a community-based medication therapy management program for asthma. Journal of the American Pharmacists Association..

[3] Oladapo AO, Rascati KL (2012). Review of survey articles regarding medication therapy management (MTM) services/programs in the United States. J Pharm Pract.

[4] Bluml BM (2005). Definition of medication therapy management: development of professionwide consensus. Journal of the American Pharmacists Association..

[5] Pellegrino AN, Martin MT, Tilton JJ, Touchette DR (2009). Medication therapy management services. Drugs.

[6] Burns A (2008). Medication therapy management in pharmacy practice: core elements of an MTM service model (version 2.0). Journal of the American Pharmacists Association..

[7] Rosenthal M, Holmes E, Banahan IIIB (2016). Making MTM implementable and sustainable in community pharmacy: is it time for a different game plan?. Res Soc Adm Pharm.

[8] Bakar ZA, Fahrni ML, Khan TM (2016). Patient satisfaction and medication adherence assessment amongst patients at the diabetes medication therapy adherence clinic. Diabetes & Metabolic Syndrome: Clinical Research & Reviews.

[9] Lim PC, Lim K (2010). Evaluation of a pharmacist-managed diabetes medication therapy adherence clinic. Pharm Pract.

[10] Alrasheedy AA, Hassali MA, Wong ZY, Saleem F (2017). Pharmacist-managed medication therapy adherence clinics: the Malaysian experience. Res Soc Adm Pharm.

[11] Ramalho de Oliveira D, Brummel AR, Miller DB (2010). Medication therapy management: 10 years of experience in a large integrated health care system. J Manag Care Pharm.

[12] Smith SR, Clancy CM (2006). Medication therapy management programs: forming a new cornerstone for quality and safety in Medicare. Am J Med Qual.

[13] Celio J, Ninane F, Bugnon O, Schneider M (2018). Pharmacist-nurse collaborations in medication adherence-enhancing interventions: a review. Patient Educ Couns.

[14] Law AV, Okamoto MP, Brock K (2009). Ready, willing, and able to provide MTM services?: a survey of community pharmacists in the USA. Res Soc Adm Pharm.

[15] Urmie JM, Farris KB, Herbert KE (2007). Pharmacy students' knowledge of the Medicare drug benefit and intention to provide Medicare medication therapy management services. Am J Pharm Educ.

[16] Herbert KE, Urmie JM, Newland BA, Farris KB (2006). Prediction of pharmacist intention to provide Medicare medication therapy management services using the theory of planned behavior. Res Soc Adm Pharm.

[17] MacIntosh C, Weiser C, Wassimi A, Reddick J, Scovis N, Guy M, Boesen K (2009). Attitudes toward and factors affecting implementation of medication therapy management services by community pharmacists. Journal of the American Pharmacists Association..

[18] Shah B, Chawla S (2011). A needs assessment for development and provision of medication therapy management services in new York City. J Pharm Pract.

[19] Jokanovic N, Tan EC, Sudhakaran S, Kirkpatrick CM, Dooley MJ, Ryan-Atwood TE, Bell JS (2017). Pharmacist-led medication review in community settings: an overview of systematic reviews. Res Soc Adm Pharm.

[20] Moczygemba LR, Barner JC, Roberson K (2008). Texas pharmacists' opinions about and plans for provision of medication therapy management services. Journal of the American Pharmacists Association..

[21] Blake KB, Madhavan SS, Scott VG, Elswick BL (2009). Medication therapy management services in West Virginia: pharmacists' perceptions of educational and training needs. Res Soc Adm Pharm.

[22] Abdullahi A, Hassan A, Kadarman N, Saleh A, Baraya YU, Lua PL (2016). Food safety knowledge, attitude, and practice toward compliance with abattoir laws among the abattoir workers in Malaysia. International journal of general medicine.

